# Assessment of an Educational Intervention to Improve Healthy Life Habits in Children Living in Vulnerable Socioeconomic Conditions

**DOI:** 10.3390/ijerph18094495

**Published:** 2021-04-23

**Authors:** María López, Irene Alcoceba, María-José Castro, María-José Cao, Sara García, Manuel Frutos, José-María Jiménez

**Affiliations:** Nursing Faculty, University of Valladolid, 47005 Valladolid, Spain; maria.lopez.vallecillo@uva.es (M.L.); irenealcoceba@hotmail.com (I.A.); mjcao@enf.uva.es (M.-J.C.); sara.garcia.villanueva@uva.es (S.G.); mafruel@enf.uva.es (M.F.)

**Keywords:** health education, schools, lifestyle, hygiene, nursing

## Abstract

Nutritional condition impacts academic performance and cognitive development. In Peru, the prevalence of chronic undernutrition in children is 6.9%, increasing the risk of mortality and morbidity. This study aimed to develop an educational intervention to achieve an improvement in the healthy habits of children in a primary education school in Lima who live in vulnerable socioeconomic conditions. We conducted a prospective quasi-experimental pre-test and post-test study of an educational intervention. The information was collected through the adaptation of the WHO questionnaire “Global School-based Student Health Survey” (GSHS), with anthropometric variables, socioeconomic level, hygiene and eating habits. One hundred eight students from 5 to 13 years old from Arenitas del Mar School in Lima (Peru) participated. The educational intervention improved eating habits. Fruit and vegetable consumption 3 or more times/day (50.9%) increased after the educational intervention (49% vs. 62.9%,) *p* < 0.0001. There was an improvement in hygiene habits, such as the frequency of handwashing with soap (32.4% vs. 63.9%) and the frequency of weekly bathing 4–6 times/week (25% vs. 47.5%) *p* < 0.0001. The educational intervention promoted better healthy living behaviors, eating habits and hygiene. This kind of initiative is a crucial tool to establish healthy living habits.

## 1. Introduction

The World Health Organization (WHO) establishes as a priority the right of children to enjoy the highest attainable standard of health. Particularly in social groups that are marginalized due to their low socioeconomic level, something that affects more than 200 million children under 5 years of age who do not reach their development potential [1]. In 2018, the under-5 mortality rate was 38.6 per 1000 children, almost half of which was due to preventable causes like easily and cheaply treatable infectious diseases [2,3].

It is estimated that by 2030, 167 million children will still be living in extreme poverty, and 3.6 million children under 5 will die that year [3]. 32% of children do not have adequate sanitation facilities, 25% have stunted growth, and 6.5% are overweight or obese, so it is essential to address this situation, including the promotion of adequate nutrition and hygienic measures to improve sanitation [4].

This problem is more pronounced in the so-called ‘‘developing” countries, such as the Latin American and Caribbean regions, where the under-5 mortality rate in 2018 was estimated at 16 per 1000 children [2]. These actions have contributed to reducing the existing gaps in the populations with less wealth and less education, although there is still a need for specific interventions in this area [5].

In Peru, 30% of children and adolescents live in poverty conditions, a situation that increases the frequency of preventable diseases and hinders children’s development [6,7].

Poor hygiene habits increase the prevalence of diseases, such as respiratory infections [8]. The 32.3% of the southern Lima district, including Villa el Salvador, live in poverty. The main cause of death is respiratory infections (17.1% in children under 5 years), with diarrhea as one of the most common pathologies [9].

Preventing potential risk behaviors, such as malnutrition or inadequate hygiene habits, is essential to promote children’s development [10], and proper handwashing can reduce the occurrence of diarrhea by 42–48% [11].

Regarding nutrition, 6.9% of children are chronically undernourished and 4.6% obese [12]. It is crucial to address malnutrition as it increases the risk of mortality and morbidity, as well as the prevalence of chronic diseases [13]. This problem also reduces academic performance and leads to stunted growth and delayed cognitive development of the child [14].

In view of this situation, educational interventions at early ages that encourage and empower children in the active management of their self-care are essential. To this end, the acquisition of healthy living habits and the control of the main risk factors should be promoted through health education programs [15]. Schools are the ideal setting to help acquire these habits at an early age, a stage in which these behaviors begin to be acquired and strengthened, to establish long-term sustainable actions with a lasting impact that favors a healthy adult life [16]. It is also important to take into account the family environment when addressing these issues, given its direct relationship to the child’s development, because it is typical of these age groups to imitate the habits of their immediate environment [17].

Several interventions have been implemented to promote healthy living habits, such as the health-promoting school programs in Lima and the Healthy Schools Program that monitors the nutritional status of school children, encourages healthy lunches and provides health education to students, parents and teachers [18,19]. Statewide, the Qali Warma school feeding program is noteworthy as it foments better nutrition for students enrolled in public educational institutions [18].

For all the above reasons, the objective of this study was to develop an educational intervention to improve the healthy habits of the children of a primary school in Lima who lived in vulnerable socioeconomic conditions.

## 2. Materials and Methods

### 2.1. Study Design

Prospective quasi-experimental pre-test and post-test study of an educational intervention in the Arenitas del Mar School in Villa el Salvador (Lima), from July to December 2019. The population under study was in vulnerable conditions due to its low socioeconomic level.

The sample size estimated to reach a 95% reliability level with a 9.5% error interval was 106 students. The province of Lima is divided into 6 metropolitan areas comprised of 43 districts. Among them, the district of Villa, located in the southern area, is the seventh poorest district in the province. 22.8% of the population (101,904 inhabitants) live in conditions of poverty. Moreover, 6.9% of children under 5 years of age are chronically malnourished, and 14.2% are overweight or obese [20].

### 2.2. Ethical Considerations

Participation in the study was anonymous and voluntary. All children who participated in the study had the informed consent form signed by their parents. The study was approved by the Clinical Research Ethics Committee of the Valladolid East Health Area (reference number: 201652).

### 2.3. Study Participants

The study was implemented on 108 primary education students between the ages of 6 and 13 at the Arenitas del Mar School. Of these, 56.4% (*n* = 61) were girls and 43.5% (*n* = 47) were boys.

The inclusion criteria were children attending Arenitas del Mar School during the 2019–2020 academic year with signed informed parental consent and voluntarily participating in the educational intervention. Students who did not have the signed parental consent or did not want to participate in the educational intervention were not included.

### 2.4. Study Measurements

The main purpose of the study was to achieve an improvement in the healthy habits of the children of the Arenitas del Mar School (Peru) using an educational intervention.

The secondary goals were to achieve an improvement in eating habits related to water intake, fruit and vegetable consumption; and to promote hygiene habits in terms of handwashing and personal hygiene.

The coordinator and collector of the surveys in the field was a member of the research team, who, at that time, was on a scholarship for International Cooperation for Development supervised by the Faculty of Nursing of the University of Valladolid at the Arenitas del Mar School in Villa el Salvador.

Before planning and developing the educational intervention, the parents of the children in the school were asked to complete an ad hoc questionnaire to identify the sociodemographic situation and health conditions of the family environment. (Table S1—Descriptive questionnaire for parents).

For the purpose of understanding the health habits that the students initially had and the impact of the educational intervention, it was conducted, before and after the intervention, the questionnaire (Table S2—CDC and GSHS adapted demographic questionnaire for children) based on the Demographic and Reproductive Health Survey conducted by the Centers for Disease Control and Prevention (CDC) [21] and the WHO Global School-based Student Health Survey (GSHS) [22].

### 2.5. Data Collection and Educational Intervention

The data collection and educational intervention were conducted at the Arenitas del Mar School and divided into four stages, as shown in Table 1.

Stage 0: Diagnosis of the situation

The educational intervention on healthy habits was explained to the school’s teaching staff, asking for their collaboration and setting up the action calendar.

There was a voluntary face-to-face meeting with the parents of students from first to sixth grade to inform them about the educational intervention, answer their questions and sign the informed consent. In this session, the parents were also asked to complete an ad hoc questionnaire to identify the sociodemographic situation and health conditions of the family environment. (Table S1—Descriptive questionnaire for parents).

Stage 1: Pre-educational intervention with the students

An anonymous and confidential questionnaire, structured in two sections, was used to assess the students’ healthy habits. In the first section, the sociodemographic information was recorded based on the Demographic and Reproductive Health Survey conducted by the CDC [21]. The variables examined were gender, age (years), weight (kg), height (m) and socioeconomic level.

In the second section, healthy habits were assessed via two categories: eating behaviors and hygiene habits, based on the WHO’s Global School-based Student Health Survey (GSHS) [22] (Table S2—CDC and GSHS adapted demographic questionnaire for children).

Stage 2: Educational intervention

The educational intervention consisted of four theoretical-practical workshops based on the “Game-Based Learning” methodology, which it is encouraged the active participation of the students. Each workshop was 60 min long, was scheduled within the school timetable and was given by a nursing student in the classrooms of the school in collaboration with the teachers responsible for each grade and with the contents approved by the academic tutor of the Faculty of Nursing. The content of the workshops was:

1. Workshop 1. We are what we eat: the importance of a balanced diet was explained, showing the food pyramid and the eating frequency of each food.
2. Workshop 2. Water as a source of hydration: It was addressed the concept of hydration, the importance of water consumption, its benefits and the impact of carbonated drinks on health.
3. Workshop 3. The importance of handwashing: It was taught how to do correct handwashing with soap, duration and steps of the procedure and its connection with disease prevention.
4. Workshop 4. We take care of our health through hygiene: The importance of oral and personal hygiene was discussed, teaching how to properly take care of ourselves.

Stage 3: Assessment of post-intervention results

The post-test questionnaire (Table S2—CDC and GSHS adapted demographic questionnaire for children) was administered to assess the impact and evaluate whether there were behavioral changes in children’s health practices. For this purpose, the questionnaire was administered to each student under the same conditions as in Stage 1.

### 2.6. Statistical Analysis

The data were analyzed using IBM SPSS v. 22.0 software (IBM, Armonk, NY, USA). Quantitative variables were expressed as mean ± standard deviation, and qualitative variables were expressed as absolute and relative frequencies (percentages). Nonparametric statistical methods were used to compare variables that did not follow a normal distribution. In the comparative analysis between qualitative variables, the chi-squared test was used. In the comparison between qualitative and quantitative variables, the Student’s *t*-test was used for one group and analysis of variance (ANOVA) for more than two groups. If they did not follow a normal distribution, the Mann-Whitney U test was used for one group and the Kruskal-Wallis test for more than one group. The significance level was determined as *p* ≤ 0.05.

## 3. Results

### 3.1. General Features of the Students and the Social and Family Environment

Table 2 shows the general characteristics of the sample by academic year and sex.

A total of 78.7% (*n* = 85) did not present chronic pathologies. Asthma was the most prevalent chronic disease (11.1%, *n* = 12), followed by language disorders (2.8%, *n* = 3). Healthcare was provided to 29.2% (*n* = 21) of students on a continuous basis and on an occasional basis 2.6 ± 1.9 times on average in the last year. Respiratory infections were the main cause of the need for it.

In the month before the survey, they missed 2.9 ± 3.1 days of school per month, with no significant differences between boys and girls. The main cause of school absenteeism in all academic years was illness (62%, *n* = 60). The first four primary grades had a higher proportion of absenteeism (73.3%, *n* = 44) than the fifth and sixth grades (26.6%, *n* = 16) *p* < 0.05.

The children lived in households with an average of 5.6 ± 3.1 people sharing the same home, and the average number of children per household was 2.5 ± 1.1. The average age of the mothers when they had their first child was 21.7 ± 4.3 years. The parents’ survey was answered almost entirely by the mothers: 95.9% (*n* = 74) were women, and 4.0% (*n* = 3) were men. 88.6% of households had running water and electricity. The mothers’ main occupation was housewife (36.1%, *n* = 39) and fathers’ most common occupation was transport sector worker (27.8%, *n* = 30) *p* < 0.05.

### 3.2. Eating Habits

The students had an average height of 1.3 ± 0.1 m and an average weight of 35.6 ± 10.8 kg. The educational intervention led to an improvement in healthy eating habits, with an increase in water consumption, a decrease in the consumption of sodas, an increase in the frequency of fruit and vegetable intake, as well as a greater commitment to breakfast at home and lunch at school (Table 3).

No statistically significant differences by gender were observed in students’ change of behavior after the educational intervention. The only exceptions were the frequency of water consumption per day, which was 1–4 glasses per day in 55.3% of boys (*n* = 26) compared to 72.1% of girls (*n* = 43) and 5 or more glasses per day in 44.7% of boys (*n* = 21) compared to 34.3% of girls (*n* = 17) *p* < 0.05.

### 3.3. Hygiene Habits

Most children before and after the educational intervention brought water from home to drink at school (80.6%, *n* = 87 VS 82.4%, *n* = 89); 86.1% (*n* = 93) of the children drank water boiled at their homes. There was no change in the type of water consumption after the educational intervention.

It was noted that after the educational intervention the number of times they brushed their teeth increased: they did not brush their teeth in the last week (19.4%, *n* = 21 vs. 19.4%, *n* = 21), 1 time per day (26.9%, *n* = 29 vs. 17.6%, *n* = 19), 2 times per day (21.3% *n* = 23 vs. 16.7% *n* = 18), 3 times per day (31.5%, *n* = 34 vs. 46.3% *n* = 50) *p* < 0.0001. Regarding the follow-up of children’s oral hygiene, there was an increase in the number of check-up visits to the dentist by 17.6%, *n* = 19 vs. 26.9%, *n* = 29 (*p* < 0.0001).

The handwashing frequency was positively modified after the educational intervention (Table 4), with an increase in handwashing with soap after touching animals on the street and when using the toilet.

The most frequent way of handwashing by children at home before eating was using tap water; this did not change after the educational intervention (79.6%, *n* = 86). All students in the school were washing their hands with water used by others in the same container, but this did not improve after the educational intervention.

There was a decrease in children, who took baths 1–3 times per week after the intervention (75%, *n* = 60 VS 52.5%, *n* = 42) compared to those who took baths 4–6 times per week (25%, *n* = 20 VS 47.5%, *n* = 38) *p* < 0.0001. The frequencies “none” (2.8%, *n* = 3) and “7 or more times per week” (23.1% *n* = 25) were not modified after the intervention.

## 4. Discussion

The healthy lifestyle practices of the Arenitas del Mar students improved after the educational intervention. Other works conducted under similar conditions addressing eating and hygiene habits [23,24,25] obtained similar results.

The impact of the educational intervention can mark the strategic direction to follow in the prevention of stunting in children of developing countries. Reducing exposure to food and hygiene risk factors using public health initiatives improves child nutrition and prevents the occurrence of infectious diseases [26].

### 4.1. Eating Habits

After the educational intervention, there was an increase in the consumption of glasses of water, albeit there was no change in the habits of the students who brought water to school. This contrasts with the work of James et al., whose educational intervention achieved an increase in the daily consumption of glasses of water by the students who brought water to school [25].

The consumption of carbonated drinks (sodas) among students is high in the whole country: 54% of them drink them daily. This high rate was reflected in our study, although it decreased after the intervention. Other studies involving interventions aimed exclusively at reducing the consumption of carbonated drinks show similar results [23,25].

The lack of vegetables and fruit in the diet is linked to a higher predisposition to chronic disease in the future [27]. Following the educational intervention, there was statistically significant growth in fruit and vegetable consumption, a similar outcome to that of other studies [28,29]. However, some studies only achieved an improvement in the consumption of vegetables and not fruit [19,30]. The use of specific strategies, such as food literacy in early childhood and school environments, has motivated children to eat fruit and vegetables [31].

Several observational studies show that, overall, between 20% and 30% of students do not eat breakfast, something that can affect their school performance and influence attention, memory and concentration [32]. This could be related to socioeconomic factors since our study showed that some of the students did not eat breakfast due to a lack of food at home. Despite this, following the educational intervention, the number of students who went to school without eating breakfast decreased. Furthermore, there was a greater commitment by the students to eat lunch at school.

Long-term studies assessing the positive effects of good eating habits on children’s growth and household food consumption patterns are still needed [33], but educational interventions, such as the one conducted in our study, will enhance better eating practices through child education that will result in improved children’s health.

### 4.2. Hygiene Habits

After the educational intervention, it was noted an improvement in hygiene habits, with similar results to those obtained by Gizaw et al. [34], that achieved an increase in the number of children with good hygiene practices after encouraging handwashing with soap in educational workshops, both after going to the toilet and after touching animals on the street. These results are in line with the studies carried out by Biran et al. [35] and Galiani et al. [24] that showed a higher prevalence of handwashing in the intervention group than in the control group. Other types of interventions that targeted the community through radio programs, advertising and events also had a positive impact on hand hygiene at both school and wider community levels [24].

Our study has revealed that sometimes the lack of means, mainly of running water, hindered improved hygiene habits, both at school and in some of the children’s homes. Thus, after the educational intervention, the children continued to wash their hands at school in the same container with water used by others. This is why institutions must be involved in addressing the socioeconomic factors because they play a role in improving healthy habits. For example, the implementation of kitchen sinks in different Peruvian communities reduced the prevalence of diarrhea and improved hygiene habits [36].

There have been several initiatives to promote healthy habits in Peruvian populations, such as the food and nutrition intervention that reduced the number of overweight children by 9.5% and obese children by 3.5% in the district of Villa el Salvador [37], and the Global Scaling Up handwashing project implemented at state-level, which consisted of a media campaign and health education interventions with very positive results in improving handwashing among the mothers and children involved [38]. The disease was identified in our work as the main cause of absenteeism. Implementing healthy hand hygiene practices will help to prevent infectious respiratory diseases, which will contribute positively to academic performance and reduce school absenteeism [24].

Following the educational intervention, there was also an increase in the number of times students brushed their teeth and the frequency of dental health checks, as described in other studies [37,38,39,40]. Students who did not brush their teeth before the educational intervention did not change this behavior after it.

Globalization and democracy are major factors in childhood health in developing countries [40], where children are more vulnerable, not only because of their biological background but also because of their economic status [41].

Currently, a sociodemographic and cultural transition is taking place with positive results for child development and growth, albeit it is conditioned by the economic level of each country, with greater progress in those countries with higher per capita income levels [42]. A global strategy for cooperation in these countries is needed to address household food insecurity and child health in low-income settings [43].

The educational intervention in a complicated environment conducted in this study has proved to be a positive strategy to promote healthy habits in children, thus presenting the opportunity to plan specific health education programs in settings.

The study’s limitations include the lack of randomness of the sample studied, which is composed of children enrolled in the Arenitas del Mar School in Villa el Salvador (Lima). It is necessary to consider the lack of a control group in the study and, therefore, other factors in addition to the intervention performed may influence the changes observed. Further limitations of the study include self-reporting of all measurements without subsequent assessment of the educational intervention in post-study stages.

## 5. Conclusions

The students manifested an improvement in healthy lifestyle habits, mainly in the increase of water consumption, fruits and vegetables, as well as in the decrease of soda consumption. Regarding hygienic habits, the students reported better handwashing and dental hygiene practices.

Educational interventions are a key tool to establish healthy living patterns, and schools are the best place to do this. It is necessary to conduct a longer follow-up of the intervention, pursuing a coordinated involvement of the teachers in the school, thus contributing to the long-term sustainability of the changes in the healthy lifestyle and hygiene habits of the students. It is also essential to achieve effective coordination through strategies that involve governments in establishing policies that contribute to health promotion, with specific interventions that actively include vulnerable populations.

## Figures and Tables

**Table 1:** Timeline of the educational intervention in Arenitas del Mar School.

	2019									
	July		August				September		December	
	Week 3	Week 4	Week 1	Week 2	Week 3	Week 4	Week 1	Week 2	Week 1	Week 2
0. Diagnosis of the situation										
1. Pre-educational intervention with the students										
2. Educational intervention										
Workshop 1—We are what we eat				Workshop 1						
Workshop 2—Water as a source of hydration					Workshop 2					
Workshop 3—The importance of handwashing						Workshop 3				
Workshop 4—We take care of our health through hygiene							Workshop 4			
3. Assessment of post-intervention results										

**Table 2:** General description of the sample by academic year and gender.

Academic Year	Gender (♀ = Girl. ♂ = Boy)		Age (Years)	*p*-Value
First Grade	♀	68.7%. *n* = 11	6.7 ± 0.4	0.06
14.8%. *n* = 16	♂	31.2%. *n* = 5	6.2 ± 0.4	
Second Grade	♀	50.0%. *n* = 8	7.0 ± 0.5	0.17
14.8%. *n* = 16	♂	50.0%. *n* = 8	7.3 ± 0.5	
Third Grade	♀	58.3%. *n* = 7	8.5 ± 0.5	0.93
11.1%. *n* = 12	♂	41.6%. *n* = 5	8.6 ± 0.5	
Fourth Grade	♀	52.1%. *n* = 12	9.6 ± 0.4	0.51
21.3%. *n* = 23	♂	47.8%. *n* = 11	9.8 ± 0.6	
Fifth Grade	♀	47.8%. *n* = 11	10.6 ± 0.6	0.38
21.3%. *n* = 23	♂	52.1%. *n* = 12	10.4 ± 0.5	
Sixth Grade	♀	66.6%. *n* = 12	11.7 ± 0.4	0.34
16.7%. *n* = 18	♂	33.3%. *n* = 6	12.0 ± 0.6	
Total	♀	56.4%. *n* = 61	9.2 ± 1.9	0.84
100%. *n* = 108	♂	43.5%. *n* = 47	9.3 ± 1.8	

**Table 3:** Comparison of students’ eating habits before and after the educational intervention.

Eating Habits		Pre-Intervention % (*n*)	Post-Intervention % (*n*)	*p*-Value
Glasses of water	None	0.9% (1)	-	<0.0001
	1 or 2 glasses/day	38.9% (42)	36.1% (39)	
	3 or 4 glasses/day	28.7% (31)	28.7% (31)	
	5 or 6 glasses/day	12.0% (13)	20.4% (22)	
	7 or 8 glasses/day	6.5% (7)	13.9% (15)	
	9 glasses or more/day	13.0% (14)	0.9% (1)	
Soda	None	16.7% (18)	16.7% (18)	<0.0001
	< 1 time/day	23.1% (25)	32.4% (35)	
	1 time/day	32.4% (35)	28.7% (31)	
	2 times/day	14.8% (16)	13.9% (15)	
	3 times/day	6.5% (7)	4.6% (5)	
	4 times/day	3.7% (4)	1.9% (2)	
	5 or more times/day	2.8% (3)	1.9% (2)	
Fruit intake	None in the last week	2.8% (3)	2.8% (3)	<0.0001
	< 1 time/day	2.8% (3)	3.7% (4)	
	1 time/day	24.1% (26)	8.3% (9)	
	2 times/day	21.3% (23)	22.2% (24)	
	3 times/day	17.6% (19)	26.8% (29)	
	4 times/day	4.6% (5)	11.1% (12)	
	5 or more times/day	26.9% (29)	25.0% (27)	
Vegetables intake	None in the last week	8.3% (9)	4.6% (5)	<0.0001
	< 1 time/day	11.1% (12)	6.5% (7)	
	1 time/day	22.2% (24)	18.5% (20)	
	2 times/day	19.4% (21)	17.6% (19)	
	3 times/day	12.0% (13)	9.3% (10)	
	4 times/day	8.3% (9)	13.0% (14)	
	5 or more times/day	18.5% (20)	30.5% (33)	
Breakfast	Never	9.3% (10)	2.8% (3)	<0.0001
	Rarely	1.9% (2)	-	
	Sometimes	13.8% (15)	19.4% (21)	
	Most days	5.6% (6)	9.2% (10)	
	Always	69.4% (75)	68.5% (74)	
Lunch at school	Never	2.8% (3)	3.7% (4)	<0.0001
	Rarely	7.4% (8)	2.8% (3)	
	Sometimes	24.0% (26)	14.8% (16)	
	Most days	7.4% (8)	6.5% (7)	
	Always	58.3% (63)	72.2% (78)	

**Table 4:** Handwashing hygiene habits comparing the impact of the educational intervention when comparing pre-test and post-test.

	Do You Usually Touch Dogs or Cats in the Street?		Do You Wash Your Hands after Touching Animals?		How Often Do You Wash Your Hands after Going to the Toilet?		How Often Do You Use Soap When You Wash Your Hands?	
	Pre-Test	Post-Test	Pre-Test	Post-Test	Pre-Test	Post-Test	Pre-Test	Post-Test
Never% (*n*)	25.9% (28)	24.1% (26)	0.9% (1)	5.6% (6)	3.7% (4)		20.4% (22)	4.6% (5)
Rarely% (*n*)	4.6% (5)	11.1% (12)	7.4% (8)	0.9% (1)	5.6% (6)		29.6% (32)	5.6% (6)
Sometimes % (*n*)	39.8% (43)	36.1% (39)	16.7% (18)	6.5% (7)	15.7% (17)	6.5% (7)	7.4% (8)	10.2% (11)
Almost always % (*n*)	7.4% (8)	8.3% (9)	15.7% (17)	15.7% (17)	17.6% (19)	10.2% (11)	10.2% (11)	15.7% (17)
Always% (*n*)	22.2% (24)	20.4% (22)	59.2% (64)	71.3% (77)	57.4% (62)	83.3% (90)	32.4% (35)	63.9% (69)
*p*-value	<0.05		<0.05		<0.05		<0.05	

## Data Availability

Not applicable.

## Supplementary Materials

The following are available online at https://www.mdpi.com/article/10.3390/ijerph18094495/s1, Table S1: Descriptive questionnaire for parents, Table S2: CDC and GSHS adapted demographic questionnaire for children.

## References

[1] Grantham-McGregor S., Cheung Y.B., Cueto S., Glewwe P., Richter L., Strupp B. (2007). Developmental potential in the first 5 years for children in developing countries. Lancet.

[2] Banco Mundial. https://datos.bancomundial.org/indicador/sh.dyn.mort?end=2018&start=1960&view=chart.

[3] Fondo de las Naciones Unidas Para la Infancia. https://uni.cf/2LnB3N4.

[4] Organización Mundial de la Salud Estrategia Mundial Para la Salud de la Mujer, el Niño y el Adolescente 2016–2030. https://bit.ly/3cC7Zh2.

[5] Popkin B.M., Adair L.S., Ng S.W. (2012). Global nutrition transition and the pandemic of obesity in developing countries. Nutr. Rev..

[6] Instituto Nacional de Estadística e Informática Evolución de l Pobreza Monetaria 2007–2018. https://bit.ly/3bqAtsp.

[7] Organización Mundial de la Salud 56ª Consejo Directivo 70ª Sesión del Comité Regional de la OMS Para las Américas. https://bit.ly/3bqllve.

[8] Ministerio de Salud de Perú (2018). Boletín epidemiológico del Perú. https://bit.ly/3dNoZAL.

[9] Ministerio de Salud de Perú (2018). Análisis de Situación de Salud. https://bit.ly/2LtIYIA.

[10] Black M.M., Walker S.P., Fernald L.C.H., Andersen C.T., DiGirolamo A.M., Lu C., McCoy D.C., Fink G., Shawar Y.R., Shiffman J. (2017). Early childhood development coming of age: Science through the life course. Lancet.

[11] Bloomfield S.F., Aiello A.E., Cookson B., O’Boyle C., Larson E.L. (2007). The effectiveness of hand hygiene procedures in reducing the risks of infections in home and community settings including handwashing and alcohol-based hand sanitizers. Am. J. Infect. Control.

[12] Navarrete P.J., Velasco J.C., Loayza M.J., Huatuco Z.A. (2016). Situación nutricional de niños de tres a cinco años de edad en tres distritos de Lima Metropolitana. Horiz. Méd..

[13] Monyeki M.A., Awotidebe A., Strydom G.L., de Ridder J.H., Mamabolo R.L., Kemper H.C. (2015). The challenges of underweight and overweight in South African children: Are we winning or losing the battle? A systematic review. Int. J. Environ. Res. Public Health.

[14] Das J.K., Lassi Z.S., Hoodbhoy Z., Salam R.A. (2018). Nutrition for the Next Generation: Older Children and Adolescents. Ann. Nutr. Metab..

[15] Mbakaya B.C., Lee P.H., Lee R.L. (2017). Hand Hygiene Intervention Strategies to Reduce Diarrhoea and Respiratory Infections among Schoolchildren in Developing Countries: A Systematic Review. Int. J. Environ. Res. Public Health.

[16] Moreno-Martínez F.J., Ruzafa-Martínez M., Ramos-Morcillo A.J., Gómez García C.I., Hernández-Susarte A.M. (2015). [Development and validation of a questionnaire on knowledge and personal hygiene habits in childhood (HICORIN®)]. Aten. Primaria.

[17] Engle P.L., Black M.M., Behrman J.R., Cabral de Mello M., Gertler P.J., Kapiriri L., Martorell R., Young M.E. (2007). Strategies to avoid the loss of developmental potential in more than 200 million children in the developing world. Lancet.

[18] Diez-Canseco F., Saavedra-Garcia L. (2017). [Social programs and reducing obesity in Peru: Reflections from the research]. Rev. Peru Med. Exp. Salud Publica.

[19] Rossi M.L., Antún M.C., Casagrande M.L., Escasany M., Ferrari M.F., Raele G., González V.B. (2019). Evaluation of the intervention of the My Healthy School Program in a cohort of schools that has participated during 2016–2017. Rev. Fac. Cienc. Med..

[20] Ministerio de Salud Análisis de Situación de Salud en Villa el Salvador. https://www.dge.gob.pe/portal/docs/asis-lima-2019/CD_MINSA/DOCUMENTOS_ASIS/ASIS_DISTRITO%20VILLA%20EL%20SALVADOR.pdf.

[21] Centros Para el Control y la Prevención de Enfermedades Cuestionario Modelo: Encuesta Demográfica y de Salud Reproductiva. https://bit.ly/2T2hdeA.

[22] Global School-Based Student Health Survey (GSHS): Core-Expanded Questions. https://bit.ly/3fOKR0v.

[23] Sichieri R., Paula Trotte A., de Souza R.A., Veiga G.V. (2009). School randomised trial on prevention of excessive weight gain by discouraging students from drinking sodas. Public Health Nutr..

[24] Galiani S., Gertler P., Ajzenman N., Orsola-Vidal A. (2016). Promoting Handwashing Behavior: The Effects of Large-scale Community and School-level Interventions. Health Econ..

[25] James J., Thomas P., Cavan D., Kerr D. (2004). Preventing childhood obesity by reducing consumption of carbonated drinks: Cluster randomised controlled trial. BMJ.

[26] Danaei G., Andrews K.G., Sudfeld C.R., Fink G., McCoy D.C., Peet E., Sania A., Smith Fawzi M.C., Ezzati M., Fawzi W.W. (2016). Risk Factors for Childhood Stunting in 137 Developing Countries: A Comparative Risk Assessment Analysis at Global, Regional, and Country Levels. PLoS Med..

[27] Micha R., Khatibzadeh S., Shi P., Andrews K.G., Engell R.E., Mozaffarian D. (2015). Global, regional and national consumption of major food groups in 1990 and 2010: A systematic analysis including 266 country-specific nutrition surveys worldwide. BMJ Open.

[28] Evans C.E., Christian M.S., Cleghorn C.L., Greenwood D.C., Cade J.E. (2012). Systematic review and meta-analysis of school-based interventions to improve daily fruit and vegetable intake in children aged 5 to 12 y. Am. J. Clin. Nutr..

[29] Sharma S.V., Markham C., Chow J., Ranjit N., Pomeroy M., Raber M. (2016). Evaluating a school-based fruit and vegetable co-op in low-income children: A quasi-experimental study. Prev. Med..

[30] Bacardí-Gascon M., Pérez-Morales M.E., Jiménez-Cruz A. (2012). A six month randomized school intervention and an 18-month follow-up intervention to prevent childhood obesity in Mexican elementary schools. Nutr. Hosp..

[31] Godrich S.L., Davies C.R., Darby J., Devine A. (2018). Strategies to Address the Complex Challenge of Improving Regional and Remote Children’s Fruit and Vegetable Consumption. Nutrients.

[32] Adolphus K., Lawton C.L., Champ C.L., Dye L. (2016). The Effects of Breakfast and Breakfast Composition on Cognition in Children and Adolescents: A Systematic Review. Adv. Nutr..

[33] Jomaa L.H., McDonnell E., Probart C. (2011). School feeding programs in developing countries: Impacts on children’s health and educational outcomes. Nutr. Rev..

[34] Gizaw Z., Addisu A. (2020). Evidence of Households’ Water, Sanitation, and Hygiene (WASH) Performance Improvement Following a WASH Education Program in Rural Dembiya, Northwest Ethiopia. Environ. Health Insights.

[35] Biran A., Schmidt W.P., Varadharajan K.S., Rajaraman D., Kumar R., Greenland K., Gopalan B., Aunger R., Curtis V. (2014). Effect of a behaviour-change intervention on handwashing with soap in India (SuperAmma): A cluster-randomised trial. Lancet Glob. Health.

[36] Hartinger S.M., Lanata C.F., Hattendorf J., Verastegui H., Gil A.I., Wolf J., Mäusezahl D. (2016). Improving household air, drinking water and hygiene in rural Peru: A community-randomized-controlled trial of an integrated environmental home-based intervention package to improve child health. Int. J. Epidemiol..

[37] Gago J., Rosas O., Huayna M., Jiménez D., Córdova F., Navarro A., Sequeiros F. (2014). Efectividad de una intervención multisectorial en educación alimentaria nutricional para prevenir y controlar el sobrepeso y la obesidad en escolares de cuatro instituciones educativas públicas del distrito de Villa El Salvador. Rev. Peru Epidemiol..

[38] Blake H., Dawett B., Leighton P., Rose-Brady L., Deery C. (2015). School-Based Educational Intervention to Improve Children’s Oral Health-Related Knowledge. Health Promot. Pract..

[39] Villanueva-Vilchis M.D.C., Alekseju-Niene J., López-Núñez B., de la Fuente-Hernández J. (2019). A peer-led dental education program for modifying oral self-care in Mexican children. Salud Publica Mex..

[40] Welander A., Lyttkens C.H., Nilsson T. (2015). Globalization, democracy, and child health in developing countries. Soc. Sci. Med..

[41] Murasko J.E. (2017). Height, BMI, and relative economic standing in children from developing countries. Am. J. Hum. Biol..

[42] Khalid H., Gill S., Fox A.M. (2019). Global aid for nutrition-specific and nutrition-sensitive interventions and proportion of stunted children across low- and middle-income countries: Does aid matter?. Health Policy Plan..

[43] Schmeer K.K., Piperata B.A. (2017). Household food insecurity and child health. Matern. Child Nutr..

