# Supplementary material for: Assessment of an Educational Intervention to Improve Healthy Life Habits in Children Living in Vulnerable Socioeconomic Conditions
Source: Int J Environ Res Public Health. 2021 Apr 23;18(9):4495. doi: 10.3390/ijerph18094495 (PMC8122981; doi:10.3390/ijerph18094495)
Supplement: Supplementary file 1 [file ijerph-18-04495-s001.zip › ijerph-1174379-supplementary.pdf]

TABLE S1- DESCRIPTIVE QUESTIONNAIRE FOR PARENTS

SOCIODEMOGRAPHIC AND HEALTH INFORMATION	
1. What is your date of birth?	
2. Gender	a) Male
	b) Female
3. How old were you when you had your first child?	
4. How many children do you have?	
5. The child who goes to school has some disease such as:	a) Respiratory disease (asthma, bronchitis...)
	b) Gastrointestinal diseases:
	c) Epilepsy
	d) Disability (Down's Syndrome)
	e) Mental Disability
	f) Learning or language delay
	g) Behavioral problems
	h) Others
6. Is your child currently in therapy or any kind of treatment?	a) Yes
	b) No
7. What treatment or therapy does your child have?	
8. How many times has your child gone to the doctor in the last year?	a) None
	b) Once
	c) 2 or 3 times
	d) 4 or 5 times
	e) 6 or 7 times
	f) 8 or 9 times
	g) 10 or more times
9. Why did he/she go to the doctor?	
10. Do any of your other children have a disease? Specify which disease:	

TABLE S2 - CDC AND GSHS ADAPTED DEMOGRAPHIC QUESTIONNAIRE FOR CHILDREN

SOCIODEMOGRAPHIC INFORMATION	
1. Year of birth	
2. Gender	a) Boy
	b) Girl
3. What grade are you in?	
4. How many people live at your home? Not including you	
5. Do you have running water and electricity in your home?	a) I have neither water nor electricity
	b) I only have water
	c) I only have electricity
	d) I have water and electricity
6. What do your parents do for work?	- Mother:
	- Father:
7. How many times have you missed school in the last month?	
8. Why did you not go to school?	a) I was sick
	b) I was grounded without going to school
	c) I was taking care of my brothers
	d) Others:
9. Do you have any diseases?	a) Asthma
	b) Diabetes
	c) Convulsions
	d) Language delay
	e) Delayed learning
	f) Anaemia
	g) Other:

EATING HABITS	
1. Height (m)	
2. Weight (Kg)	
3. How many glasses of water do you drink in a day?	a) None
	b) 1 or 2 glasses
	c) 3 or 4 glasses
	d) 5 or 6 glasses
	e) 7 or 8 glasses
	f) 9 or more glasses
4. How many times a day do you usually drink soda?	a) None
	b) Less than once a day
	c) 1 time per day
	d) 2 times a day
	e) 3 times a day
	f) 4 times a day
	g) 5 or more times a day
6. How many times a day do you usually eat fruit?	a) None in the last week
	b) Less than once a day
	c) 1 time per day
	d) 2 times a day
	e) 3 times a day
	f) 4 times a day
	g) 5 or more times a day
7. How many times a day do you usually eat vegetables?	a) None in the last week
	b) Less than once a day
	c) 1 time per day
	d) 2 times a day
	e) 3 times a day
	f) 4 times a day
	g) 5 or more times a day
8. How often do you eat breakfast?	a) Never
	b) Rarely
	c) Sometimes
	d) Most days

	e) Always
9. During the last week, did you bring lunch to school?	a) Never
	b) Rarely
	c) Sometimes
	d) Most days
	e) Always

HYGIENE HABITS	
1. How many times a day do you usually brush your teeth?	a) None
	b) 1 time a day
	c) 2 times a day
	d) 3 times a day
	e) 4 or more times a day
2. The last time you went to the dentist, what was the main reason?	a) I have never been to the dentist
	b) Something was wrong with my teeth or gums
	c) To follow up on a treatment from a previous visit
	d) For a check-up
3. Do you usually touch dogs or cats in the street?	a) Never
	b) Rarely
	c) Sometimes
	d) Almost always
	e) Always
4. Do you wash your hands after touching animals?	a) Never
	b) Rarely
	c) Sometimes
	d) Almost always
	e) Always
5. How often do you wash your hands after going to the toilet?	a) Never
	b) Rarely
	c) Sometimes
	d) Almost always
	e) Always
6. How often do you use soap when you	a) Never

wash your hands?	b) Rarely
	c) Sometimes
	d) Almost always
	e) Always
7. How do you usually wash your hands before eating at school?	a) I do not usually wash my hands
	b) In a container with water used by others
	c) In a container with water used only by myself
	d) Under the tap water
	e) Otherwise:
8. How do you usually wash your hands before eating at home?	a) I do not usually wash my hands
	b) In a container with water used by others
	c) In a container with water used only by myself
	d) Under the tap water
	e) Otherwise:
9. How many times do you usually bathe per week?	a) None
	b) 1 time
	c) 2 times
	d) 3 times
	e) 4 times
	f) 5 times
	g) 6 times
	h) 7 times or more
10. How do you normally drink the water at home?	a) Bottled water
	b) Tap water
	c) Boiled water
	d) I don't drink water
